# Magnifying Networks for Histopathological Images with Billions of Pixels

**DOI:** 10.3390/diagnostics14050524

**Published:** 2024-03-01

**Authors:** Neofytos Dimitriou, Ognjen Arandjelović, David J. Harrison

**Affiliations:** 1 Maritime Digitalisation Centre, Cyprus Marine and Maritime Institute, Larnaca 6300, Cyprus; 2 School of Computer Science, University of St Andrews, St Andrews KY16 9SX, UK; oa7@st-andrews.ac.uk; 3 School of Medicine, University of St Andrews, St Andrews KY16 9TF, UK; david.harrison@st-andrews.ac.uk; 4 NHS Lothian Pathology, Division of Laboratory Medicine, Royal Infirmary of Edinburgh, Edinburgh EH16 4SA, UK

**Keywords:** histology, histopathology, deep learning, whole slide image, digital pathology, gigapixel images, tissue heterogeneity, precision medicine

## Abstract

Amongst the other benefits conferred by the shift from traditional to digital pathology is the potential to use machine learning for diagnosis, prognosis, and personalization. A major challenge in the realization of this potential emerges from the extremely large size of digitized images, which are often in excess of 100,000 × 100,000 pixels. In this paper, we tackle this challenge head-on by diverging from the existing approaches in the literature—which rely on the splitting of the original images into small patches—and introducing magnifying networks (MagNets). By using an attention mechanism, MagNets identify the regions of the gigapixel image that benefit from an analysis on a finer scale. This process is repeated, resulting in an attention-driven coarse-to-fine analysis of only a small portion of the information contained in the original whole-slide images. Importantly, this is achieved using minimal ground truth annotation, namely, using only global, slide-level labels. The results from our tests on the publicly available Camelyon16 and Camelyon17 datasets demonstrate the effectiveness of MagNets—as well as the proposed optimization framework—in the task of whole-slide image classification. Importantly, MagNets process at least five times fewer patches from each whole-slide image than any of the existing end-to-end approaches.

## 1. Introduction

One of the most practically important examples of image analysis with billions of pixels can be found in digital pathology and, in particular, in the task of whole-slide image (WSI) classification [1,2]. WSIs are digitized microscope slides that are stored in a multi-resolution pyramid format, with the original high-resolution image at the top level and progressively downsampled versions beneath. A typical WSI at the highest resolution is about 100,000 × 100,000 pixels, which amounts to approximately 50 GB of uncompressed data. The shift to automated feature learning with neural networks (NNs) has greatly advanced image analysis, but the sheer size of WSIs presents unique challenges.

Although other computer vision approaches have been introduced in recent years (e.g., using transformer architectures [3]), convolutional neural network (CNN)-based methodologies still constitute one of the most effective and popular choices as a way of automatically learning image features rather than handcrafting them [4,5,6]. CNNs typically excel in the processing of images with a size of fewer than one million pixels [7,8,9,10,11]. Although some recent work has explored the use of higher resolution images (e.g., up to 8192×8192 [12]), CNN-based learning directly from images with billions of pixels is not possible due to computational and memory constraints. Instead, the majority of existing approaches first subdivide a WSI into smaller images, that is, patches that can be directly used as CNN input [13,14,15,16,17,18,19,20,21,22,23,24]. However, these computationally demanding pipelines inevitably impose an *a priori* belief on the magnification scale, field of view, and location of each of the extracted patches [25]. Such constraints run the risk of introducing errors as their assumptions are often violated, and then real-world practice deviates from these assumptions.

In response to the described outstanding challenges to the existing methods, we introduce a new family of neural networks, henceforth referred to as magnifying networks (MagNets). MagNets learn to use an attention-based mechanism to decide (on a recursive, coarse-to-fine basis) the regions of the gigapixel image that are most likely to provide useful information after being analyzed at a finer scale. Incidentally, this is conceptually similar to a pathologist’s knowledge and attention-based use of magnification with a brightfield microscope. Importantly, as we show experimentally, the MagNet models can be optimized without the need for extra supervision for the attention layers. As a result of this optimization, patches from varying magnification scales, fields of view, and locations are extracted.

**Figure 1:**
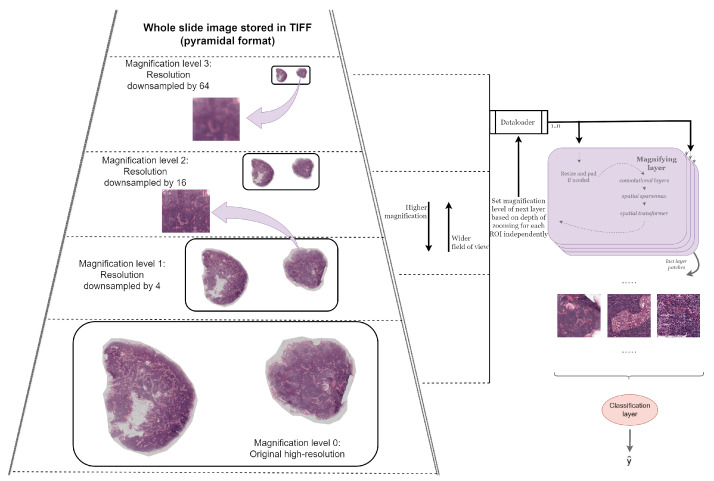
An illustration of the interaction between a MagNet and a WSI. The illustrated WSI has four magnification levels, with the original high-resolution image being magnification level 0, and each of the versions beneath is progressively downsampled by four. As visualized, for the same ROI, magnification level 3 is blurry when compared to magnification level 1. The depicted MagNet model consists of four magnifying layers and a classification layer. DataLoader accesses the right magnification level for each ROI independently based on the depth of zooming so far. Note that the ROIs of the last layer can span across different magnification levels and with varying levels of fidelity, thereby providing information across multiple resolutions and multiple fields of view.

In order to benefit from weakly supervised learning, that is, the lack of need for overly laborious manual annotation, a MagNet employs two key strategies. First, it has an end-to-end design where each magnifying layer feeds into the next, ultimately leading to a classification layer at the end. Secondly, within each magnifying layer, comprising a spatial transformer module [26] and convolutional layers, the network computes the affine transformations from an input image of size I×I and, subsequently, applies them to a version that is not only of higher resolution, $I^{\prime }\times I^{\prime }$ (e.g., $I^{\prime }=2\times I$), but is also potentially sourced from a higher magnification level, as depicted in Figure 1. At a high level, MagNets provide a novel way of solving both the “where” (i.e., the identification of salient information within a WSI) and “what” (i.e., a visual understanding of salient information) problems of gigapixel image analysis in an end-to-end fashion [1].

MagNets offer a superior alternative to the patch-based approaches of gigapixel image analysis, especially in the context of digital pathology, as they come with innate transparency (embedded hard attention), no preprocessing requirements (i.e., end-to-end training capability with gigapixel images), and an ability to perform both localization and classification tasks with no additional information (only slide-level information is used). Our key contributions are the following:

- In the context of the WSI classification of metastases, we propose the possibility of identifying and magnifying ROIs starting from a very-low-resolution downsampled version of the WSI (three channels; 56×56 pixels), and, experimentally, we show that recursively identifying and magnifying regions of interest (ROI) allows for the extraction of informative areas across magnification levels.
- Without leaving the weakly supervised paradigm, we explore nested attention using the spatial transformer module for gigapixel image analysis.
- To the best of our knowledge, this is the first work that automatically learns to select regions that are analyzed at potentially progressively greater magnification levels and, thus, fuses extracted information across scales. As such, the proposed method is able to exploit rich contextual and salient features, overcoming the typical problem of patch-based processing that poorly captures the information that is distributed beyond the patch size.

## 2. Related Work

In this section, we concentrate on the deep learning-based methods addressing the “where” problem in tissue slide analysis, as this is where the key novelty and strength of MagNets lie. Importantly, we emphasize that methods addressing the complementary “what” problem are compatible with and can be readily integrated within the MagNet model. Solutions to the “where” problem involve modeling the spatial distribution of salient information within a tissue slide [19] such that more targeted processing can be enabled—one that does not depend on the analysis of the entire gigapixel image. The challenge, often referred to as the “what” problem, emerges from the need to identify visual patterns that are salient to the task at hand [19]. An example would be the learning of a visual representation of cancer cell morphology by a neural network as a means of classifying tumor vs. nontumor regions. There is a large body of work that proposes novel ways of addressing the “what” problem, such as by incorporating contrastive loss [14], task-specific self-supervision [27], or using better, pretrained networks [17].

### 2.1. Patch Extraction

#### 2.1.1. Strongly Supervised

One way of identifying and extracting relevant information from gigapixel images relies on the use of annotations from domain experts. More specifically, for WSIs, patches based on annotations by a pathologist can be extracted in such a way as to ensure a balanced training dataset. A relatively large body of work exists that follows this paradigm [27,28,29,30,31,32,33,34]. Most of these approaches extract the ROI from a single magnification level, e.g., the largest available at 20× or 40×. A few, such as the approach of Sui et al. [34], extract patches from annotated areas at multiple magnification levels instead.

However, the fully-supervised nature of these approaches limits their applicability to many clinical tasks for which annotating to this extent is either extremely laborious and expensive or simply infeasible (e.g., cancer prognosis) [35].

#### 2.1.2. Weakly Supervised

In the absence of pixel-level annotations but with the availability of slide-level ground truth, the literature is divided into three main methods of tackling the “where” problem. The most prominent approach is to tile the entirety of a WSI region that satisfies certain simple and predefined low-level criteria, such as those based Otsu’s thresholding, entropy, or color [13,14,15,16,17,18,19,20,21]. The second approach involves random sampling from a grid-like patch population [22,23]. Methodologies that use either of the above two approaches need to mitigate the large number of extracted patches in the later parts of their pipelines. For example, a few recent works have employed instance-level self-supervision under the multi-instance learning paradigm to mitigate the highly unbalanced nature of tiling [14,17].

However, there are still at least two key drawbacks of the aforementioned approaches. Firstly, the field of view, i.e., the visible area of the tissue slide in a patch, is not optimally selected, and, therefore, objects of interest may not fit within a single image patch. Secondly, the predefined locations of the patches (e.g., from a grid) may lead to objects of interest being split across patch boundaries. More closely related to our methodology is the third approach, which does not suffer from these drawbacks, as it utilizes attention modules to select and extract the most informative patches.

### 2.2. Patch Selection

#### 2.2.1. Attention

BenTaieb and Hamarneh [36] employed a recurrent visual attention network that finds sub-regions of interest within a tiled WSI (with each tile having a size of 5000 × 5000 pixels). Notably, there is no upsampling mechanism, and the patches are predefined as non-overlapping tiles. Furthermore, only one magnification scale was utilized. Qaiser and Rajpoot [37] used an attention network on images with 1024 × 1024 pixels at the 2.5× magnification scale to identify, extract, and process patches from higher, predefined magnification scales (10× or 20×). Both approaches, along with a number of others [38,39,40], are nondifferentiable and, therefore, can only be optimized using reinforcement learning or variational methods [41] rather than backpropagation. Recent work, however, has turned to differentiable alternatives [38,41,42,43].

#### 2.2.2. Nested Attention

None of the approaches thus far can be employed on gigapixel images directly [44]; instead, patch extraction based on predefined preprocessing is required. Kong and Henao [44] were the first to introduce the concept of nested attention, and by extending the attention module introduced by Katharopoulos and Fleuret [41], proposed a two-layer hierarchical attention model that enables the end-to-end training of deep learning models from WSIs. Although conceptually similar, MagNets further extend the idea of nested attention by allowing an arbitrary number of attention layers (called magnification layers herein) and by not enforcing any *a priori* assumptions on the selected patches (Katharopoulos and Fleuret [41] enforce a non-overlapping grid over what can be extracted from a WSI).

## 3. Materials and Methods

### 3.1. Datasets

The Camelyon datasets contain WSIs from the surgically resected lymph nodes of breast cancer patients [4,45]. These WSIs were independently curated across multiple hospitals. Camelyon16 includes images from 238 normal and 160 cancerous tissue sections, whereas the publicly available portion of Camelyon17 has a total of 500 WSIs (318 normal and 182 cancerous) [46]. In addition, in the case of metastasis, metadata is available as to the extent of the metastasis (macrometastasis, micrometastasis, or isolated tumor cells (ITCs)). Since only a few cases contain the much more difficult ITC type of metastasis (36 cases, i.e., ≈4% of all cases), it is unlikely that they are sufficiently representative of the ITC class. Therefore, they are excluded from the training dataset.

We follow the protocol described in the Camelyon competition website [34] and, in addition, set aside 25% of Camelyon17 as a testing set (36 with ITC, 17 with micro-, and 20 with macrometastasis, i.e., in total, 73 WSIs with metastases and 88 WSIs of normal tissue). We shuffle the remaining WSIs from Camelyon17 with the Camelyon16 WSIs and train on the 80% portion, validating the better models from the remaining 20%. The best MagNets (based on the validation set) are retrained on both the training and validation data and are evaluated on the testing set. Since ITC cases were excluded and the models were trained for WSI classification rather than patient-level pN prediction, the MagNet models were not evaluated on the privately held testing set.

The pixel-level annotations that are available for some of the WSIs are only used in the post-processing analysis of the models. During training, we only use the binary slide-level label that indicates the presence, or lack thereof, of cancerous cells within the gigapixel image.

### 3.2. Magnifying Networks

A MagNet consists of *L* magnifying layers followed by a classification layer. The magnifying layers are responsible for identifying the information relevant to the task at hand at a specific magnification level and extracting it in the form of multiple image patches. Each image patch is sourced from a larger image version of the WSI and one that is potentially from a higher magnification level, i.e., more fine-grained details can appear. The classification layer is concerned with the visual understanding of the extracted patches.

#### 3.2.1. Magnifying Layer

**Figure 2:**
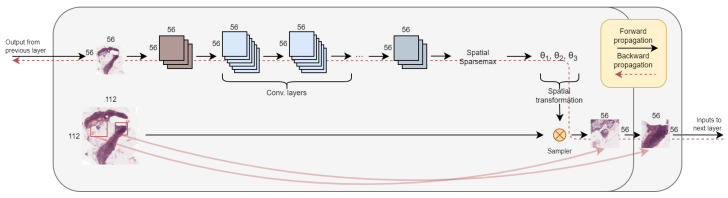
An illustration of a single magnifying layer that outputs two patches. The convolutional layers are independent between the two patches. The red squares illustrate the affine transformation based on the outputted thetas. Note that if this was the last magnifying layer, the image size of the patches would have been 224×224.

An illustration of a single magnifying layer is shown in Figure 2. We now explain its structure and function in detail.

##### Resizing and Padding

As we subsequently employed convolutional layers, expecting 56×56-pixel images, input *I*—either as a single input image or a set of images—is resized to $56\times h_{i}$ or $w_{i}\times 56$ based on bi-linear interpolation, with the smaller side, $h_{i}$ or $w_{i}$, then symmetrically padded (new pixels are black to match the filter) so that $h_{i}=56$ or $w_{i}=56$ accordingly. For the purpose of up-sampling (explained in detail shortly: see the “Sampling” paragraph), a larger version $I^{\prime }$ (112×112 pixels) is also generated using the same protocol.

Note that although preliminary experiments were conducted using larger images as input to the magnifying layers (*I* and $I^{\prime }$ with 112×112 and 224×224 resolutions, respectively), the training of the MagNets with more than two magnifying layers on a single GPU was not feasible at these resolutions.

##### Convolutional Layers

**Figure 3:**
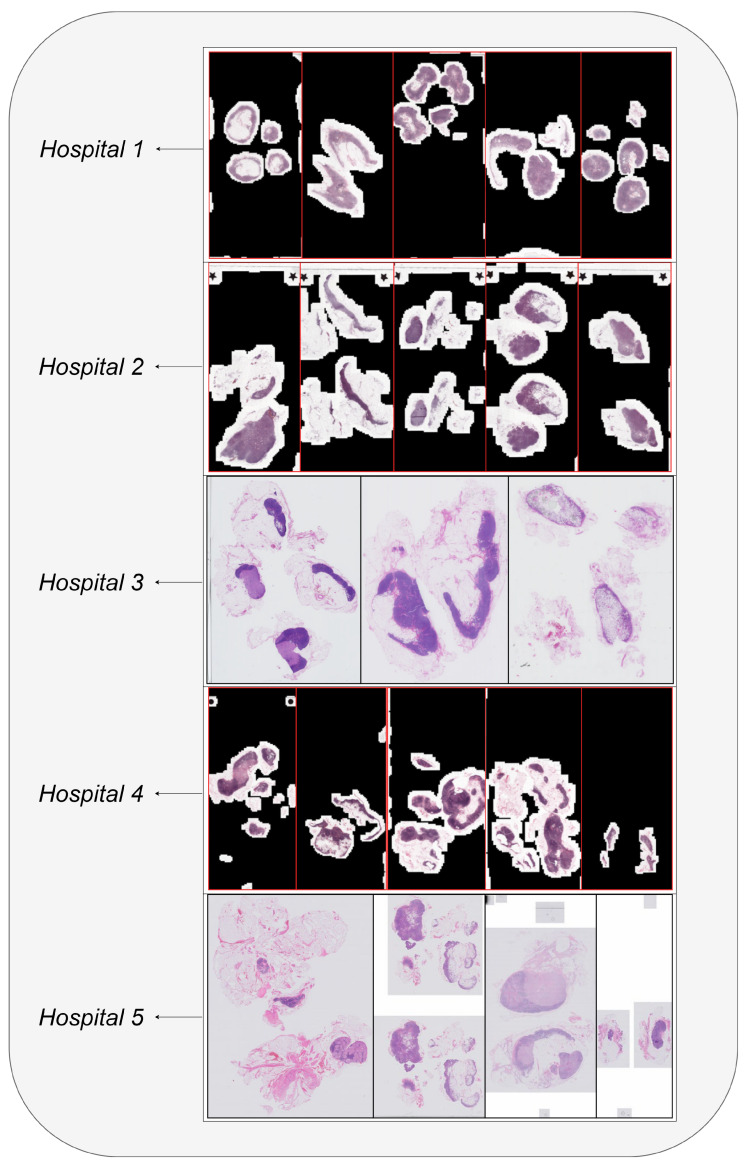
Randomly sampled WSIs from each hospital.

The salient regions in each image patch vary significantly in size. This comes as a consequence of the varying levels of metastasis but also from the lack of standardization in WSI digitization across different institutes and scanners (see Figure 3).

Therefore, the right kernel size for the convolutional operations varies depending on *I*. Hence, we stack convolution layers with different kernel sizes similarly to InceptionNet-v3 [47].

Let *Conv2D* be a n×n convolution layer (with the padding set to 1), followed by batch normalization and ReLU nonlinearity. *MaxPool* is a max pooling operation with a 3×3 kernel and padding. We define a “Branch” as the simultaneous forward pass of the input through five layers, where a *layer* sequentially applies a number of *Conv2D* and *MaxPool* operations. In particular, the five layers are the following:

- 1×1 *Conv2D*;
- 1×1 *Conv2D*⇝3×3 *Conv2D*;
- 1×1 *Conv2D*⇝3×3 *Conv2D*⇝3×3 *Conv2D*;
- 1×1 *Conv2D*⇝3×3 *Conv2D*⇝3×3 *Conv2D*⇝3×3 *Conv2D*;
- *MaxPool*⇝1×1 *Conv2D*.

The outputs of all of the layers above are concatenated into a single tensor. Since padding is employed, the output has the same height and width as the input. MagNets use patch and layer-specific “Branches”, e.g., a two-layer MagNet with two patches extracted, and each magnifying layer has six of these layers (two at the first layer and four at the second).

**Figure A1:**
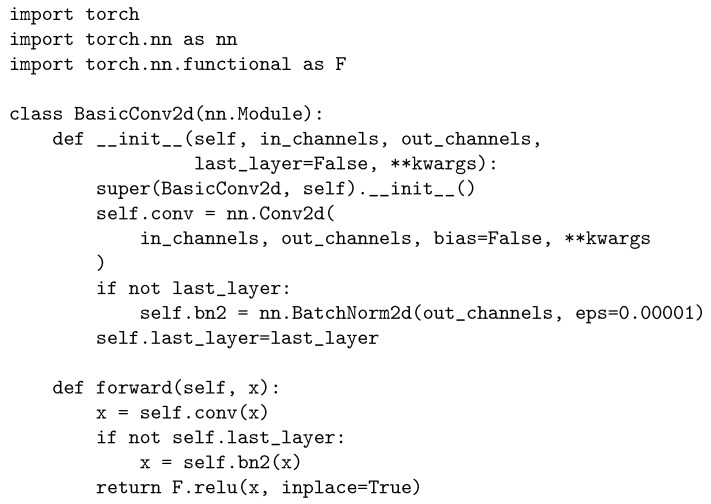
PyTorch implementation of *Conv2D*.

**Figure A2:**
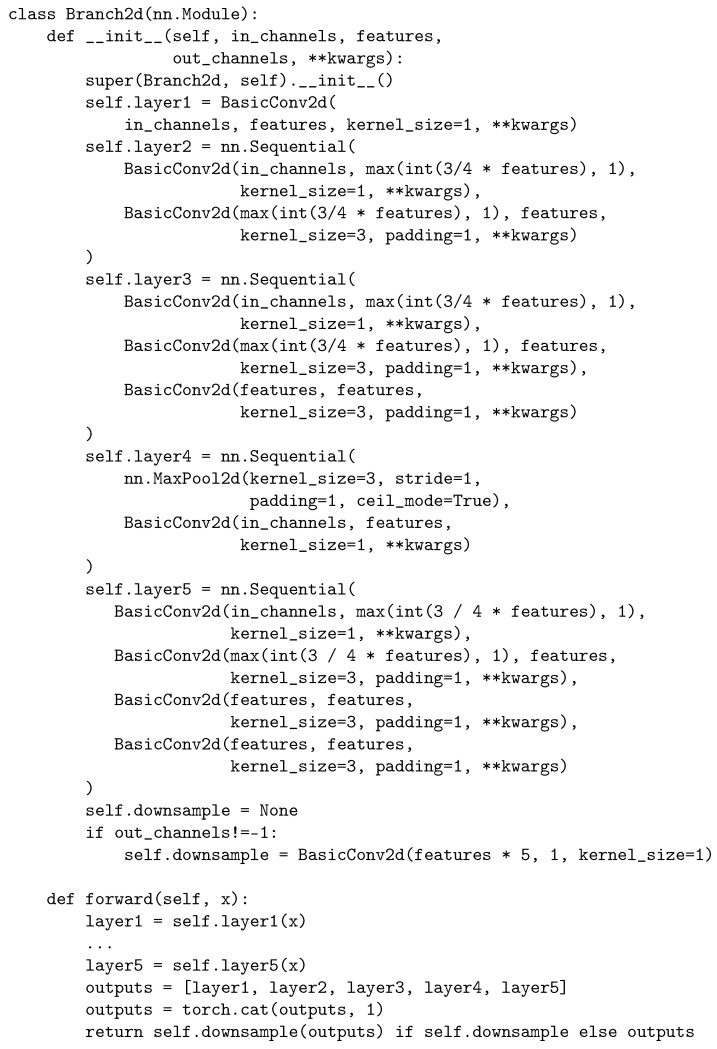
PyTorch implementation of a *Branch*.

A more elaborate description, as well as the pseudocode, of *Conv2D* and *Branch* is provided in Figure A1 and Figure A2, respectively.

##### Spatial Transformer

A spatial transformer network (STN) is used to transform hard attention-based cropping into a differentiable process. An STN consists of three parts: a localization network, a grid generator, and a sampler [26].

The *localization network* in the literature is typically a fully connected or recurrent neural network [48] that receives an input from a CNN, and its role is to output a spatial transformation of the co-ordinate space of the original image [38]. However, due to their high demand for GPU VRAM owing to their large number of parameters, both options are impractical for employment within MagNets. Instead, MagNets utilize a spatial sparsemax in the last convolutional layer, for which the output can be used to infer the spatial transformation (hard attention-based cropping) parameters (*s*, $t_{x}$, $t_{y}$) directly. In particular, the dimensions of the output of the last convolutional layer are the same as the input image, i.e., 56×56 pixels. Following the application of the spatial sparsemax operation, the output can be thought of as a probability mass function with the expected L1 norm translating to the scaling parameter (*s*), and the translation parameters ($t_{x}$ and $t_{y}$) obtained by the expected value for the indices of the *x*-axis and *y*-axis, respectively.

Given the transformation parameters *s* for isotropic scaling and $t_{x}$, for $t_{y}$ for translation along each axis, we further constrain the parameters as follows:

(1) $$\begin{array}{cc} s & =max(s,0.05) \end{array}$$

(2) $$\begin{array}{cc} t_{x} & =tanh(t_{x}) \end{array}$$

(3) $$\begin{array}{cc} t_{y} & =tanh(t_{y}) \end{array}$$

with θ of spatial (affine) transformation $A_{\theta }$:

(4) $$\begin{array}{c} \theta =\left[\begin{array}{ccc} s & 0 & t_{x}\\ 0 & s & t_{y} \end{array}\right] \end{array}$$

**Figure A3:**
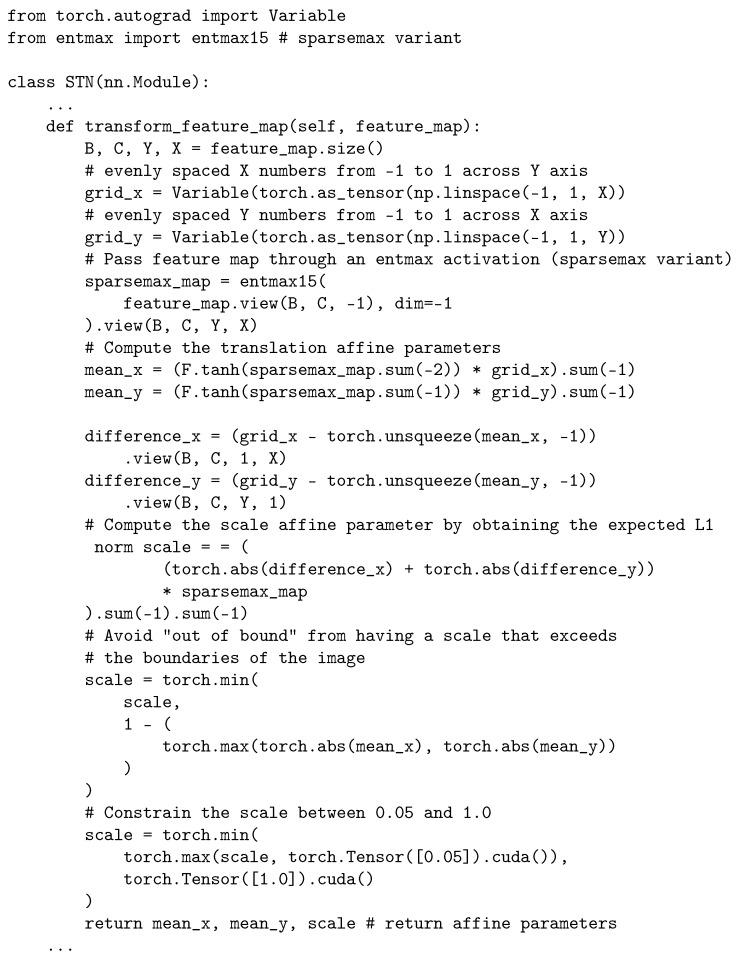
PyTorch implementation of inferring the affine parameters from a given feature map for the STN.

The tanh constraint on the translation parameters implicitly forces the network to favor center extraction, whereas the minimum bound imposed on the scaling helped experimentally with the vanishing gradients within STs during the early stages of training. An implementation is provided in Figure A3.

The *grid generator* then creates the desired grid by multiplying θ with a 56×56-pixel meshgrid. Finally, an image can be interpolated onto the grid using a *sampler*.

##### Sampler

**Figure 4:**
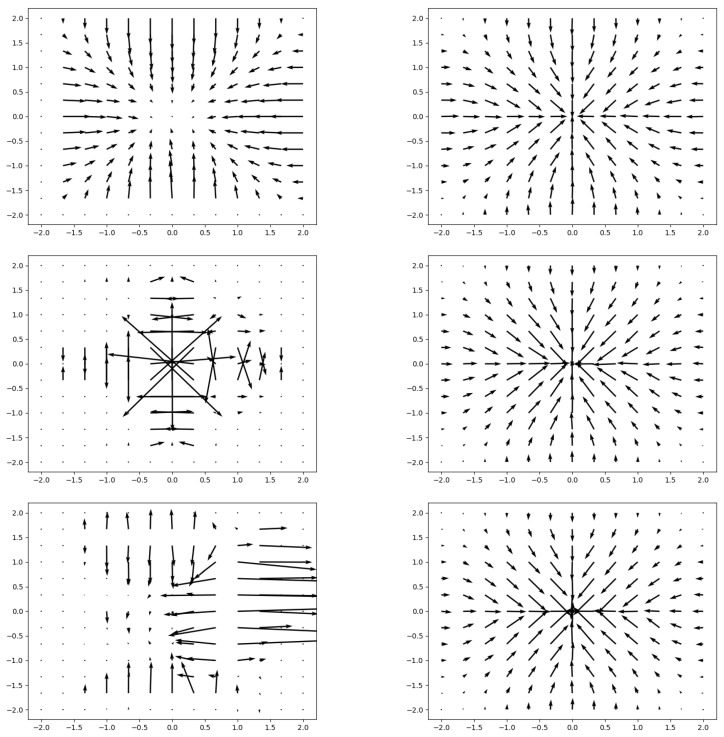
Gradient analysis of using bi-linear sampling (**left**) vs. the linearized multi-sampling approach (**right**) [49]. From top to bottom, the image is not downsampled, downsampled by a factor of 4, and downsampled by a factor of 8.

A sampler takes a set of sampling points along with an image and applies a differentiable sampling kernel to produce the sampled image. Bi-linear interpolation is a poor choice for a sampling kernel for our work, as shown in the empirical analysis we present in Figure 4 (conducted on the training set), which is also supported by the literature; it performs poorly under severe scale changes [49], with poor gradient propagation. Wei et al. [49] proposed an alternative sampler, Linearized multi-sampling, the gradients of which are resilient to the amount of scaling. We use the original implementation of this sampler provided by Wei et al. [49].

##### Sampling

This is the part that makes each layer “magnifying”. MagNet applies the transformation $A_{\theta }$ on $I^{\prime }$ instead of *I*, thereby allowing the output to contain information (finer-grain) that was potentially not present in *I*. An example of a magnifying layer that outputs two patches is shown in Figure 2. By stacking multiple magnification layers together, MagNets are able to retrieve information from increasingly higher magnification levels.

The magnification level from which $I^{\prime }$ is extracted is set dynamically, as illustrated in Figure 1. In particular, given $h_{0}$, $w_{0}$ has the height and width of a WSI (at the highest magnification level, i.e., pyramid level 0), and $h_{c}$ and $w_{c}$ are the height and width of a requested ROI (based on the affine transformation of the STN); the magnification level *m* is calculated as follows:

$$\begin{array}{c} R_{h}=\left\lfloor log_{2}\left(\frac{h_{o}}{h_{c}}\right)\right\rfloor ,\\ R_{w}=\left\lfloor log_{2}\left(\frac{w_{o}}{w_{c}}\right)\right\rfloor ,\\ R=max(R_{h},R_{w}),\\ m=max(m_{max}-R,0) \end{array}$$

where $m_{max}$ is the total number of magnification levels of the WSI. For example, given a WSI of 50,000 × 100,000 pixels and nine magnification levels, access to a specific magnification level depends on the requested area (width × height) as per the following:
Width
HeightWSI resolutionlevel≥25,000and≥50,000171×391 pixels8≥12,500or≥25,000391×782 pixels7≥6250or≥12,500782×1563 pixels6≥3125or≥62501563×3125 pixels5≥1563or≥31253125×6250 pixels4≥782or≥15636250× 12,500 pixels3≥391or≥78212,500 × 25,000 pixels2≥171or≥39125,000 × 50,000 pixels1<171and<39150,000 × 100,000 pixels0

The above assumes that the spatial resolution of the WSI is halved at each subsequent magnification level, which is, indeed, the case for the Camelyon dataset.

#### 3.2.2. Classification Layer

At the last magnifying layer, the images to be forwarded to the classification layer are sampled using a grid with a 224×224 pixel resolution (instead of 56×56 pixels). These images are passed through an ImageNet pretrained CNN (InceptionNet-v3) that outputs a feature map into a gated recurrent unit (GRU) network. The output of the GRU is passed through an FCNN (two layers with 512 and 256 hidden neurons, respectively) to output a slide-level $\hat{y}$ estimate, i.e., whether the given WSI contains cancer or not.

#### 3.2.3. Auxiliary Classifiers

A form of both self-supervision and weak-supervision is introduced by using two auxiliary classifiers. These are ImageNet-pretrained ResNet-18 networks [8] that output a slide-level prediction using the extracted images from magnifying layer 1 and layer 3, respectively.

Firstly, paradoxical loss was employed as a form of self-supervision [39]. The premise of paradoxical loss is that information presented for the layer-3 images should provide an equally good or better prediction than that from layer 1. Under this assumption, instances where the opposite is observed are viewed as “undesirable and paradoxical” [39]. The paradoxical loss over *M* inputs is computed as follows:

$$L_{1}=\frac{1}{M}\sum_{i=0}^{M}max\left(P_{1}-P_{3},0\right),$$

where $P_{1}$ and $P_{3}$ are the estimated probabilities of identifying the true class label (slide-level) by using patches from layers 1 and 3, respectively.

In addition, a cross-entropy loss, $L_{2}$, is used between the slide-level label *y* and the ResNet-18 outputs, $\hat{y}$, as a form of weak supervision. The binary cross-entropy loss $L_{2}$ over *M* labels and outputs is defined as follows:

$$L_{2}=\frac{1}{M}\sum_{i=0}^{M}\left[y_{i}\log\left({\hat{y}}_{i}\right)+\left(1-y_{i}\right)\log\left(1-{\hat{y}}_{i}\right)\right].$$

#### 3.2.4. Configurations

A MagNet consists of *L* magnifying layers, each of which can access increasingly higher magnification scales, as determined (dynamically) from the degree of zoom (i.e., *s*) thus far. At each layer, *l*, $P_{l}$ number of patches are extracted (ROI).

A consequence of the recurrent nature of MagNets is that an exponential number of patches are extracted and analyzed from a single gigapixel image if more than one patch is extracted per layer. In particular, given a constant *P* across the layers, we see the following:

$$\mathrm{Total}\mathrm{patches}\mathrm{extracted}\mathrm{in}\mathrm{layer}l=\left\{\begin{array}{cc} l, & \mathrm{if}\;P=1.\\ P^{l}, & \mathrm{otherwise}. \end{array}\right.$$

We find that extracting two ROIs in some magnifying layers and three ROIs in others provides a balance between a sufficient rate of expansion (breadth) while allowing for up to four-layer MagNets (depth) to be trained on a GPU with 24 GB of VRAM. The effectiveness of this configuration is corroborated by the ablation experiments summarized in Table 1.

### 3.3. Evaluation

#### 3.3.1. Data Augmentation

For any given image (both during training and inference), we apply a filter that sets grey image pixels (including the degenerate form of grey that is white) as black. In particular, these are pixels for which the corresponding red, green, and blue channel values differ from each other by less than 15 (scale 0–255). This filter removes the background and various scanning artifacts (smudges, etc.) that are most strongly visible in the otherwise nearly uniform regions of the slide. We employ neither color normalization nor random color perturbation [28,50]. We find the latter to be ineffective [51], whereas the former is avoided since it would add significant computational overhead to WSI analysis. The synthetic data augmentation we performed during training involves horizontal and vertical mirroring and rotations by 90, 180, and 270 degrees.

#### 3.3.2. Training

The final networks were trained using the Adam optimizer [52] for 200 epochs. For the hyperparameter tuning and ablation experiments, the models were trained for 20 epochs. A batch size of 16 and 8 was employed for the MagNet networks with three and four layers, respectively. The initial learning rate was set to $3\times 10^{-5}$ and was decayed using a cosine annealing scheduler [53]. All ResNet-18 and InceptioNet-v3 networks were ImageNet-pretrained networks. The ST convolutional layers were randomly initialized.

#### 3.3.3. “Frozen” Patch

Differences in the clinical pipelines leading to the creation of WSIs, e.g., due to different scanning profiles (see Figure 3), result in significant differences between the WSIs of different hospitals. For example, some hospitals process WSIs so that they only contain regions with tissue, whereas others do not (Hospital 1 vs. Hospital 3 in Figure 3). In order to mitigate the above variance, we freeze the first patch of the second layer so that it always attends to the whole input image. This allows for the image to catch up in quality in the cases where a large amount of zooming was required at the first magnifying layer, e.g., for a WSI with small areas of tissue or one that was not preprocessed and depicts the entire tissue slide.

#### 3.3.4. Loss Functions

We employ the paradoxical loss function ($L_{1}$) as a form of self-supervision for the convolutional layers within the STNs. In addition, cross-entropy is used between the slide-level labels and both of the last outputs of the GRU ($L_{3}$), as well as the ResNet-18 outputs($L_{2}$). $L_{1}$ and $L_{2}$ are described in Section 3.2.3, and $L_{3}$ is the same as $L_{2}$, except that $\hat{y}$ represents the GRU outputs. The final loss function is computed as the sum of $L_{1}$, $L_{2}$, and $L_{3}$.

## 4. Results

**Figure 5:**
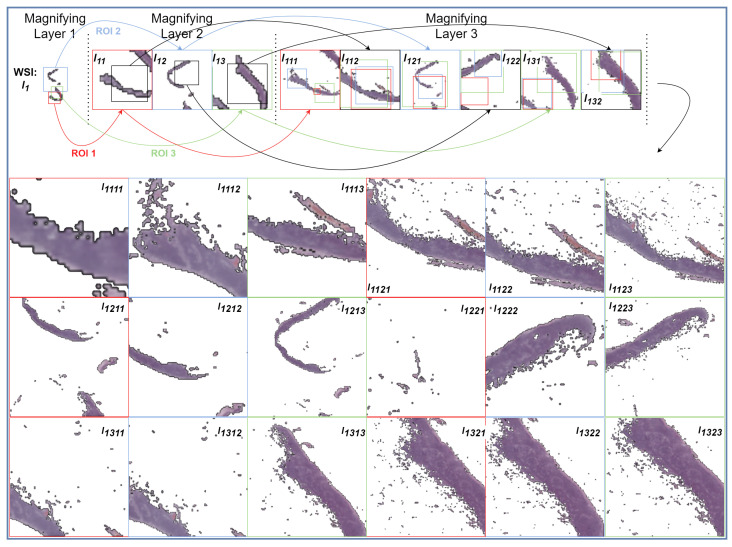
A visualization of a forward pass of a WSI $I_{1}$, with micro-metastasis (from the testing set) passed through a three-layer MagNet model. The background of the images is shown in white for visualization purposes. In the first magnifying layer, three ROIs can be identified from $I_{1}$, namely, $I_{11}$ (red outline), $I_{12}$ (blue outline), $I_{13}$ (green outline), each of which was forwarded to the second magnifying layer. In the second magnifying layer, two ROIs can be identified for $I_{11}$, $I_{12}$, and $I_{13}$, resulting in six forwarded images to the third magnifying layer. Finally, three ROIs can be identified in the last magnifying layer for each forwarded image, resulting in 18 images being forwarded to the classification layer. Each of these 18 images can be traced backward based on their annotated name, e.g., $I_{1311}$ is the first ROI (red outline) of $I_{131}$, which is the first ROI (red outline) of $I_{13}$, which, finally, is the third ROI (green outline) of $I_{1}$. Note how the images forwarded to the classification layer have a 224×224×3 resolution rather than 56×56×3.

In order to evaluate the proposed method, by using the optimization framework described in the previous section, we trained three-layer and four-layer MagNets for the task of cancer metastasis detection from WSIs. For ease of the comparative analyses, we included a baseline encoder, as reported by Tellez et al. [19], based on average color intensity (termed RGB baseline), as well as the dual-stream multiple instance learning network (DSMIL) [14] and the two-stage hierarchical attention sampling method (HAS) [44], as evaluated on the micro- and macro-metastases of Camelyon16. The DSMIL constitutes one of the most competitive methods in the weakly supervised paradigm, but it involves extensive preprocessing steps, namely, the extraction of millions of patches at different magnification scales [14]. On the other hand, HAS has no preprocessing steps. Nevertheless, contrary to MagNets, HAS requires a large number of patches to be dynamically extracted from each attention layer (50–100), with each layer specific to a predefined magnification scale (as selected prior to training), and each patch loci predefined in a grid-like fashion. We hypothesize that MagNets are able to solve the “where” problem more efficiently than HAS due to the lack of such constraints, e.g., see Figure 5. A summary of the results is presented in Table 2, which shows the AUROC—the standard evaluation metric used in the related literature [4,19,29,45]—and the accuracy (the threshold was set to 0.5).

The three-layer MagNet model processes 28 image patches per WSI, comprising 10 image patches with 56×56×3 pixels and 18 with 224×224×3 pixels, i.e., a total of ≈3 million pixels processed per WSI. The four-layer MagNet model processes 28 images at a 56×56×3-pixel resolution and 36 at a 224×224×3-pixel resolution, totaling approximately 6 million pixels per WSI. In comparison, the competing method, HAS, samples 100 images in the first stage (100×100×3 pixels each) and 50 to 100 in the second stage (400×400×3 pixels each), processing approximately 27 to 51 million pixels per WSI [44]. The DSMIL-LC method requires the processing of more than 1 billion pixels per WSI, with an average of 625 and 8000 image patches at 5× and 20× magnification scales, each at a 224×224×3-pixel resolution [14]. Therefore, the MagNet models demonstrate a significant reduction in the number of pixels processed per WSI by a factor of at least five compared to the most efficient existing approach.

When evaluated on the testing set, the three-layer MagNet model distinguished macro-, micro-, and ITC metastases vs. normal cases with AUROC/accuracy scores of 95/88%, 71/78%, and 57/69%, respectively. For the macro- and micro-metastases alone, i.e., with ITC cases excluded, the model achieved an AUROC of 84% and an accuracy of 77%. Across all case types, it averaged an AUROC of 71% and an accuracy of 64%. For the four-layer MagNet model, the performance on the testing set showed an AUROC/accuracy of 91/89%, 76/83%, and 63/70% for the macro-, micro-, and ITC metastases, respectively. When considering the macro- and micro-metastases, the model’s AUROC was 84%, with an accuracy of 81%. The aggregated performance across all case types resulted in an AUROC of 75% and an accuracy of 66%. In comparison, the HAS method reportedly achieved an accuracy of 83% when differentiating macro- and micro-metastases from normal cases. The DSMIL-LC approach exhibited superior performance on the same task, with an AUROC of 90% and an accuracy of 92%.

**Figure 6:**
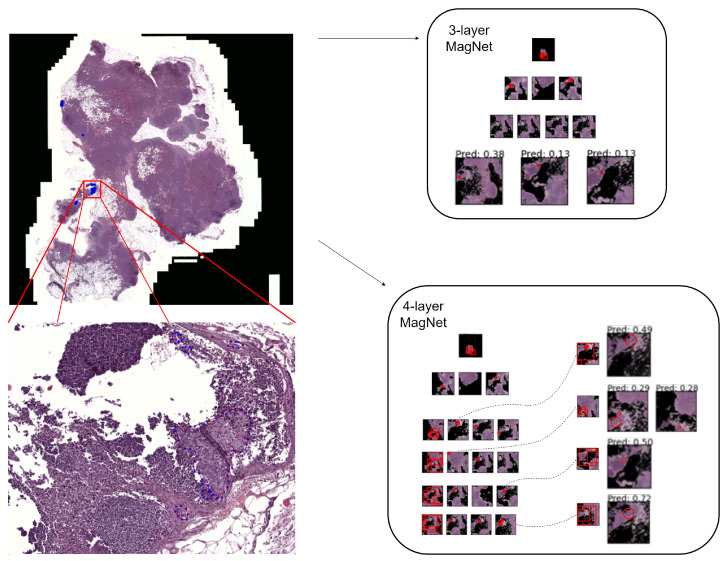
A WSI from Hospital 3, wherein the four-layer MagNet correctly identified a cancerous region, whereas the three-layer MagNet produced a false negative.

We re-evaluated the final MagNet models (both the three-layer and the four-layer versions) against the *testing set* of WSIs, but this time, we took the different hospitals that the WSIs came from into consideration. There are (15,11,3,4), (13,7,6,2), (19,2,2,4), (14,8,6,3), and (16,8,0,7) cases for normal tissue, ITC, micro-metastases, and macro-metastases, respectively, for Hospitals 1 to 5 (see examples from the different hospitals in Figure 3). A summary of the results is provided in Table 3. We computed the ranking capabilities (AUROC) between the normal and tumor cases (scanned using MagNets) from the same hospital for all five hospitals independently. No or minimal discrepancy is observed between the performance of the models for Hospitals 1, 4, and 5. However, the four-layer MagNet performs better than the three-layer MagNet on micro-metastasis cases from Hospital 2 (54% vs. 85%), and for the cases from Hospital 3, the opposite is observed, i.e., the three-layer MagNet performs better (95% vs. 58%). For Hospital 2, with six micro-metastasis cases, the false negatives (i.e., the classification of a WSI showing cancer as being normal) from the three-layer MagNet was the source of the discrepancy. The extra magnifying layer of the four-layer MagNet provided higher resolution images that, for the above cases, were needed for the cancer to appear in the patches. In Figure 6, we show an example of a WSI that was incorrectly classified as negative by a three-layer MagNet but was correctly classified as positive by a four-layer MagNet, together with explanatory visualizations corresponding to the two networks. For Hospital 3, the discrepancy came down to the decision of one case with micro-metastasis (Hospital 3 only had two micro-metastasis cases). The four-layer MagNet misses the part of the WSI that had cancer from the very first magnifying layer, whereas the three-layer MagNet correctly classifies it.

We also investigate the scaling that is typically learned by the four-layer MagNet across the different hospitals and different cases (normal vs. different types of mestastases). No major difference is observed between layers 2, 3, and 4, with the average scale learned being 0.5. Nevertheless, the standard deviation ranges (significantly) from 0.1 to 0.3. For layer 1, a mean scale difference is observed between the different hospitals, with the average and standard deviation being 0.21±0.13, 0.31±0.15, 0.32±0.22, 0.25±0.13, and 0.32±0.22 for Hospitals 1 to 5 in order. Since there are no universal scanning settings (see Figure 3, e.g., Hospitals 1, 2, and 4 scanned the whole tissue slide, whereas Hospitals 3 and 5 applied a form of cropping in most cases), the shift in the mean scale seems to be the model’s approach to generalizing across different hospitals.

Finally, the inclusion of loss functions $L_{1}$ and $L_{3}$ and the “frozen” patch, as well as the specific MagNet configuration (i.e., patches per layer), was supported by the outcomes of the ablation studies shown in Table 1.

## 5. Discussion

**Figure 7:**
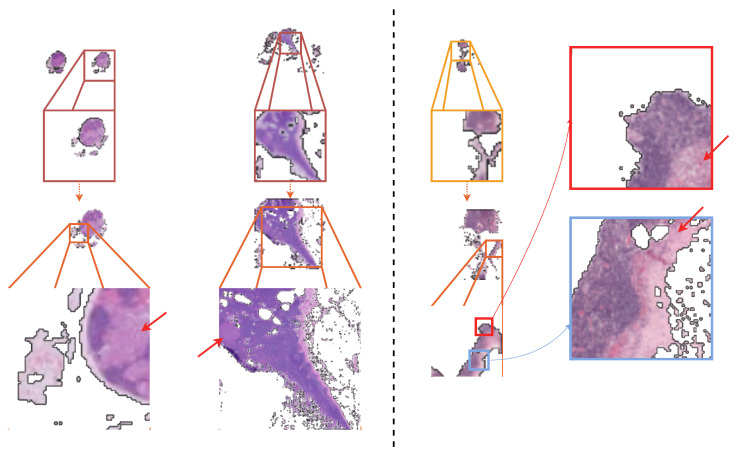
The cancerous regions of macro- and micro-metastasis, as identified by the three-layer MagNet model (on the left), and the micro-metastasis identified by the four-layer MagNet (on the right). The pointing red arrows show the cancer regions based on the annotations provided by the pathologists at the highest magnification scale.

MagNets exhibit robust and effective exploration capabilities, namely attending to image content in an attention-driven manner, exploring WSIs at the various magnification levels best suited to the task at hand, and learning how to fuse relevant information both within the same WSI region and across different regions and magnification levels. In addition, the classifier (in the form of InceptionNet) demonstrates an excellent ability to distinguish normal from cancerous tissue across samples, irrespective of the magnification scale. The examples corroborating this are shown in Figure 7.

MagNets do not require WSIs to be patch-based preprocessed. Instead, the network (starting from the lowest magnification level) can dynamically explore a WSI at the continually higher magnification levels that the MagNet sees as fit in the visual context of the specific WSI. The premise is that the patches with the best magnification level, field-of-view, and location— according to the optimizing task—will be dynamically extracted. Indeed, for the task of breast cancer metastasis detection, our MagNet models performed extremely well, given that they only had 28 to 64 image patches per WSI to process from—far less than any of the existing approaches (processing at least five times fewer pixels per WSI [14]). Furthermore, the MagNet models demonstrate robust generalization capabilities, evidenced by their good performance in ITC cases (even in the absence of ITC examples during training) and their adaptability to scan diverse profiles across different hospitals.

### Limitations

The Camelyon dataset provides a fitting optimization challenge for MagNets, which is that of breast cancer metastases detection, considering the varying granularity that needs to be assessed when predicting macro-metastases, micro-metastases, and isolated tumor cells (ITCs) from WSIs. However, it could be argued that the configurations of MagNet that were explored herein are not adequate for clinical adoption. In particular, due to the unconstrained and non-exhaustive nature of exploration, a MagNet could miss a region containing a metastasis early on, thus producing a false negative. With clinical adoption in mind, more exhaustive exploration would be required by perhaps increasing the number of patches that are extracted at each magnifying layer. Moreover, although the Camelyon datasets are useful and are, indeed, the most appropriate public corpora for the evaluation of MagNets on the task of gigapixel image analysis, it is important to appreciate that they were collected for a very specific set of analytical tasks, namely, the localization of tumor regions and the holistic classification of WSIs as being cancerous or not. However, the above results cannot guarantee the same efficacy for MagNets when applied to a problem where the “identification of patches when zoomed-in” is not as clear-cut. Moreover, since the hospital of origin of each WSI was not considered while creating the testing set, it is possible that the generalization of the trained models may not extend beyond these five hospitals.

## 6. Conclusions

In this work, we introduced the MagNet—a neural network consisting of fully-connected, convolutional, and recurrent layers that employ STs in a novel manner so as to facilitate attention and data-driven recurrent exploration and, ultimately, end-to-end learning from gigapixel images. The built-in hard attention mechanism of MagNets makes them well-suited for clinical use. In particular, the explanations generated by MagNets are visually intuitive, e.g., as shown in Figure 7, for a domain-specific expert to interpret (as they visually depict a subset of the original WSI) and can be generated “on the go” without any additional overhead.

Crucially, the efficiency of MagNets regarding gigapixel images is unparalleled, mitigating the high GPU memory demands typically associated with gigapixel image analysis. This attribute of MagNets holds particular promise for deployment in clinical settings, where computational efficiency translates to cost-effectiveness and practicality, especially in scenarios where GPU availability is a limiting factor. Furthermore, the capability to process vast images with limited hardware resources opens the avenue for implementing deep learning-based services on mobile and edge devices, significantly expanding the reach and accessibility of advanced diagnostic tools.

Finally, MagNets can be optimized without extra supervision (e.g., by further bounding box annotations) for the task at hand. This is of high significance since, for most clinical tasks, collecting the ground truth data required for a higher degree of supervision is either extremely laborious and expensive or simply not possible, e.g., in the case of patient prognosis.

## Figures and Tables

**Table 1:** Ablation experiments for the different components of a three-layer MagNet using three random seeds and random training-validation splits. AUROC decreases when a smaller number of patches is used and when $L_{2}$, $L_{3}$, or the “Frozen” patch is omitted.

# Patches	$L_{2}$	$L_{3}$	Frozen Patch	AUROC [%]
3, 2, 3	🗸	🗸	🗸	68.8
2, 2, 2	🗸	🗸	🗸	63.1
2, 3, 2	🗸	🗸	🗸	66.1
3, 2, 3	🗸		🗸	67.3
3, 2, 3		🗸	🗸	68.0
3, 2, 3			🗸	66.9
3, 2, 3	🗸	🗸		62.4
3, 2, 3				62.9

**Table 2:** The results of the MagNet models on the testing set against the baselines and existing competitive methods for the classification of macro and micro-metastases vs. normal cases. The number of pixels processed per WSI reflects the computational efficiency of each method.

Method	# of Pixels Processed per WSI	AUROC [%]	Accuracy [%]
Mean RGB Baseline [19]	-	58	-
DSMIL-LC [14]	>1 billion	90	92
HAS [44]	27 to 51 million	-	83
3-layer MagNet	≈3 million	84	77
4-layer MagNet	≈6 million	84	81

**Table 3:** The results of our MagNet models on the WSI subsets of the testing set (the percentages correspond to AUROC), sorted by their hospital of origin.

Three-Layer MagNet	Macro- and Micro-	Macro-	Micro-	All
Hospital 1	89%	95%	80%	73%
Hospital 2	60%	77%	54%	58%
Hospital 3	96%	97%	95%	91%
Hospital 4	87%	98%	81%	68%
Hospital 5	-	92%	-	79%
**Four-Layer MagNet**	**Macro-**	**Micro-**	**Macro- and Micro-**	**All**
Hospital 1	88%	97%	84%	76%
Hospital 2	85%	77%	85%	74%
Hospital 3	84%	97%	58%	78%
Hospital 4	75%	93%	65%	68%
Hospital 5	-	92%	-	79%

## Data Availability

Data are available in a publicly accessible repository. The data presented in this study are openly available in multiple repositories, as listed here (accessed 29 February 2024): https://camelyon17.grand-challenge.org/Data/ [45].

## Appendix A. Implementation Details

MagNets and the optimization framework were implemented using the following packages in Python: PyTorch [54], OpenSlide [55], Scikit-Image [56], Numpy [57], matplotlib [58], Pillow [59], and scikit-learn [60]. The sparsemax implementation was adopted from https://github.com/deep-spin/entmax [61] (accessed 29 February 2024). The attention layer was inspired by the implementation from https://github.com/TolgaOk/Differentiable-Hard-Attention-Module (accessed 29 February 2024).

### Appendix A.1. Convolutional Layers

The first *Branch* takes an input with three features (e.g., an image) and outputs a tensor with 15 features, i.e., each of the five layers outputs tensors with three features. The second *Branch* takes the output of the first *Branch* and outputs a tensor with 40 features (eight from each of the five layers). In the third *Branch*, given the input of the second *Branch*, one feature is extracted from each of the five layers, and the concatenated output is forwarded through a 1×1 *Conv2D* that returns a tensor with one feature. Given that the input images in the experiments had a 56×56 pixel resolution, the output from the third *Branch* is a tensor with 56×56×1 dimensions. The implementation of *Conv2D* and *Branch* are shown in Figure A1 and Figure A2, respectively.
